# Utilization of Buccal Free Gingival Graft to Restore the Peri‐Implant Soft Tissue Texture and Color Match in the Esthetic Zone: A Case Report With 3 Years Follow‐Up

**DOI:** 10.1002/ccr3.70781

**Published:** 2025-08-06

**Authors:** Neda Moslemi, Mahdie Rahami, Mohammad Shokri, Hossein Khoshkhou, Amir Raee

**Affiliations:** ^1^ Department of Periodontology, School of Dentistry Tehran University of Medical Sciences Tehran Iran; ^2^ School of Dentistry Tehran University of Medical Sciences Tehran Iran; ^3^ Department of Periodontology School of Dentistry, Guilan University of Medical Sciences Tehran Iran; ^4^ Department of Periodontology School of Dentistry, Mashhad University of Medical Sciences Tehran Iran

**Keywords:** autografts, dental implants, esthetic, gingiva, oral surgery

## Abstract

This case report represents soft tissue management around a dental implant in the upper lateral incisor area in a young female with a high esthetic demand and excessive gingival display. During implant placement, a soft tissue augmentation utilizing connective tissue graft was conducted to augment the interproximal papilla around the implant. However, the coronally flap advancement resulted in mucogingival distortion and a lack of a sufficient amount of keratinized mucosa around the dental implant. In order to achieve an aesthetically acceptable color and texture match, a free gingival graft (FGG) was harvested from the buccal gingival tissue in the premolar site instead of the palatal site. In the follow‐up visits, the mucosa around the implant was quite comparable to the gingiva around the adjacent teeth. It seems that the “buccal gingiva” can be considered as the first choice of donor site for FGG in the esthetic zone.

**Summary**

- Buccal free gingival grafting demonstrates its potential utility in selected esthetic zone cases requiring vestibular depth restoration and soft tissue management regarding texture/color matching.
- The presented technique shows promising results in this specific clinical scenario, though further controlled studies are needed to validate its efficacy.

## 1 Introduction

For a long time, implant dentistry was focused on hard tissue quality and quantity as the main prerequisite for implant success. However, recent evidence has demonstrated the importance of stable and healthy soft tissue from the biological and esthetic perspectives [1]. A common complication, called midfacial peri‐implant soft tissue dehiscence, has been associated with limited keratinized tissue width and mucosal thickness [2]. Moreover, the esthetic demand of the patients has progressively increased, and even a minimal discrepancy in the level of mucosal margin is considered unacceptable [3].

Most often, implants in the esthetic area need either soft tissue or hard tissue augmentation, which is routinely accompanied by buccal flap advancement to cover the graft [4, 5, 6, 7]. However, one of the main consequences after buccal flap advancement is the loss of keratinized mucosa and vestibular depth, which may result in mucosal color and texture mismatch around the dental implant compared to the adjacent natural teeth [8, 9].

Several techniques have been described for the restoration of keratinized mucosa and vestibular depth [10, 11, 12, 13, 14, 15, 16].

The roll technique was introduced by Abrams in 1980 to thicken the volume of soft tissue in the buccal aspect [17]. This technique is used to uncover the implant fixture in the second‐stage surgery and at the same time increase the buccal thickness of the mucosa around the implant. The original Abrams' roll technique has been modified and revisited by several authors to overcome the limitations [18, 19]. However, this technique is not relevant in cases with a complete lack of buccal keratinized tissue.

The apically displaced flap is one of the surgical techniques to increase the keratinized mucosa around the dental implants in the second‐stage surgeries [20, 21]. However, due to the presence of the rugae in the anterior palate, this technique may not be acceptable in the anterior maxilla from an esthetic perspective.

The masticatory mucosa of the palate is a wonderful source for harvesting autogenous grafts. The classic free gingival graft (FGG) from the palate is the most well‐documented and predictable technique to increase the keratinized mucosa and vestibular depth around dental implants [22]. Due to the different characteristics of the palatal epithelium compared to that of the buccal side, the color and texture of the classic FGG is a challenging issue in the esthetic zone [23]. Therefore, this technique is not recommended in the esthetic zone [24]. Some modifications have been suggested to mitigate the FGG's unfavorable aspects [25, 26, 27]. However, to the best of our knowledge, none of them demonstrated ideal results. In a study by Urban et al. in 2020, they utilized a labial strip gingival graft (LGG) to reconstruct severely distorted mucogingival defects. However, they placed the LGG in the apical area in combination with connective tissue graft or xenogenic collagen matrix in the coronal area [26]. In the present case report, we placed a buccal FGG in the marginal mucosa, which was not stripped, and included both the keratinized gingiva and the alveolar mucosa. Herein, we present a modification of buccal FGG for the management of soft tissue deficiency around a single anterior implant in a high‐esthetic‐demand patient with a high smile line profile to restore the color and texture of the peri‐implant marginal mucosa.

## 2 Case History

Complete written informed consent was obtained from the patient for the publication of this study and the associated images. This case report was registered and verified by the ethical committee with the following registration number; IR.TUMS.DENTISTRY.REC.1403.047.

A 26‐year‐old female was referred to the periodontics department for replacement of the upper right lateral incisor (tooth #7) with a dental implant. The tooth had been lost 6 months ago because of extended caries and failure of endodontic therapy. The patient was a nonsmoker and in good systemic and periodontal health. She had high expectations for the esthetic outcome and presented with a gingival display and a high smile line.

**FIGURE 1:**
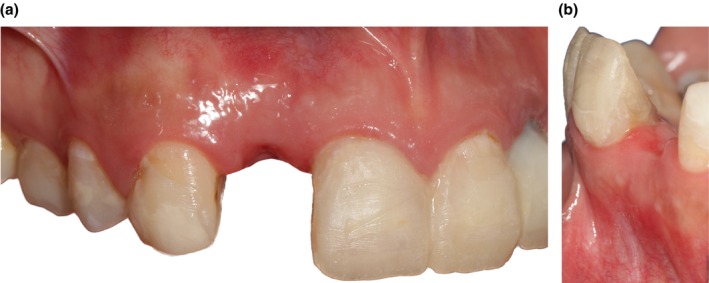
(a) The clinical baseline profile. The loss of papilla was noted in the mesial of the edentulous site. (b) Baseline edentulous contour. The concave buccal contour was observed.

Clinical examinations revealed that the gingival phenotype was thin, the mesial papilla in the edentulous site was lost (Figure 1a); and the buccal contour was concave (Figure 1b).

The treatment plan was based on digitally guided implant placement and simultaneous peri‐implant plastic surgery to reestablish the lost soft tissue in height and thickness.

### 2.1 Investigations and Treatment

#### 2.1.1 First Surgical Intervention (Implant Placement and Modified Connective Tissue Platform Technique)

**FIGURE 2:**
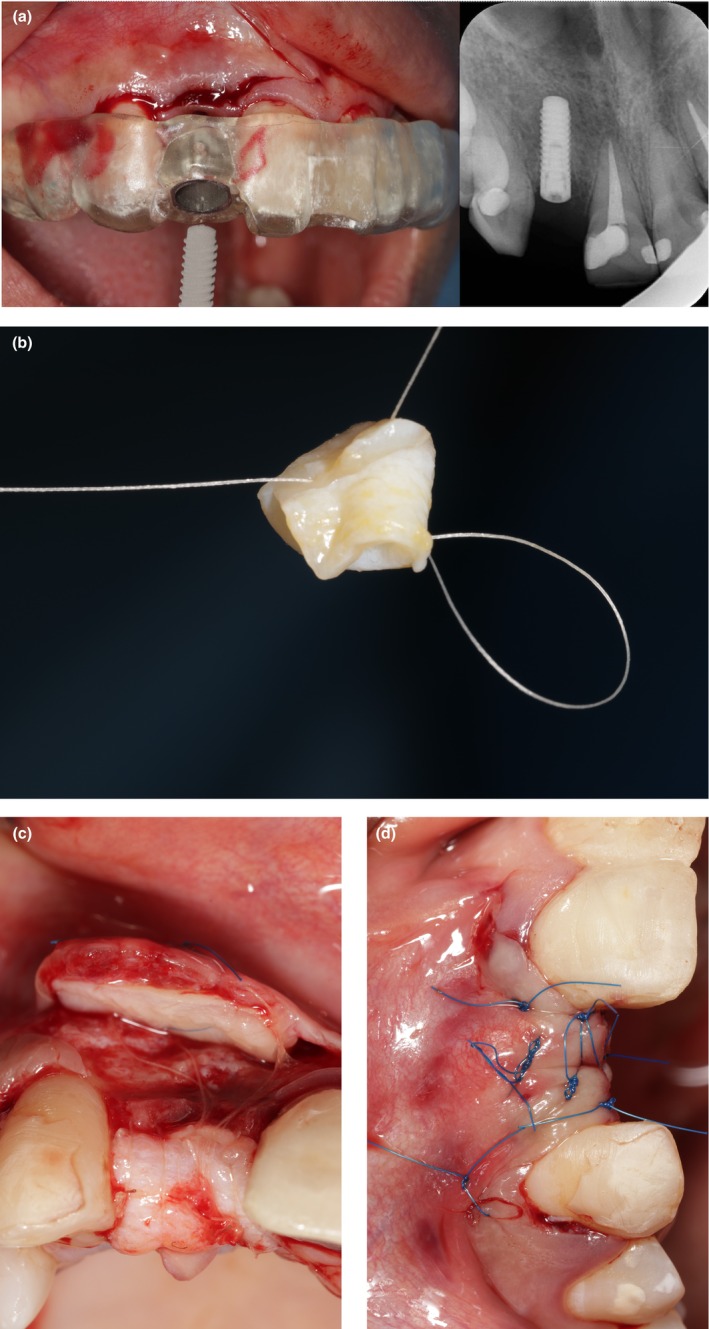
(a) Implant Insertion. A Straumann implant 3.3 × 10 mm was inserted using a digitally designed guide, which is depicted in periapical radiography. (b) Crestal de‐epithelized FGG. A rolled de‐epithelized FGG was attached using a PGA 6.0 suture to enhance the papilla height at the crest. (c) Buccal de‐epithelized FGG. The second part of the de‐epithelialized FGG was securely fixed using PGA 6.0 through single interrupted suturing to the underlying periosteum in the buccal area. (d) Flap closure. The flap was closed with nylon 6.0 and interrupted sutures.

After delivery of local anesthesia, the surgical guide was firmly placed over the maxillary adjacent teeth. A tissue punch was used to make a circular incision. The punched tissue was carefully removed using a microsurgical tweezer. According to the standard protocol of the manufacturer, drilling was performed through the surgical guide. A 3.3 * 10 BL implant (SLA, Institute Straumann AG, Basel, Switzerland) was inserted (Figure 2a) and a cover screw was placed. A flap with two vertical incisions was raised in the buccal area, and the modified connective tissue platform technique [28, 29] was used to augment the peri‐implant soft tissue in the vertical and horizontal directions (Figure 2b–d). The original connective tissue platform technique [28] uses a folded connective tissue graft in the buccal and a connective tissue graft in the occlusal; however, we did it vice versa. Following the preparation of the recipient bed, a long FGG was harvested from the palate. The graft was de‐epithelialized and divided into two parts. One part was folded and sutured with PGA 6.0 suture (Figure 2b) and then fixed on top of the crest area with the same suture material. The second part of de‐epithelialized FGG was fixed with PGA 6.0 in the buccal area with a single interrupted suturing technique to the underneath periosteum (Figure 2c). Then, mattress sutures were used to adapt the graft to the underlying bed. The flap was coronally advanced and sutured tension‐free, with nylon 6.0 coronally by horizontal internal mattress sutures in combination with the single interrupted sutures to achieve complete closure (Figure 2d).

Sutures were removed 14 days after the surgery. After 3 weeks, a provisional fixed prosthesis supported by the adjacent teeth was delivered.

**FIGURE 3:**
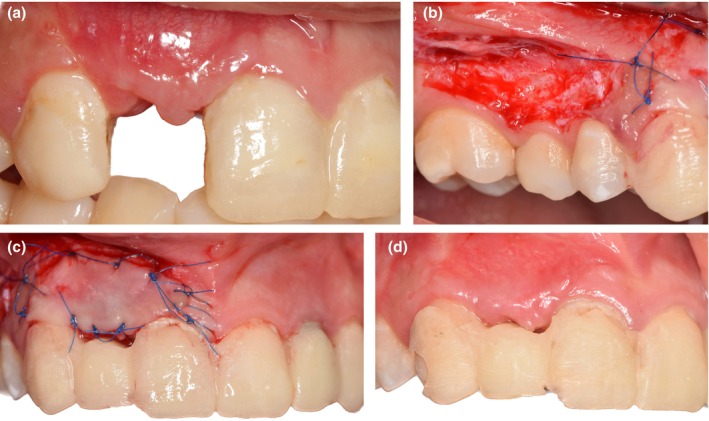
(a) Clinical view at 2 months; decision making about performing a free gingival graft (FGG). The soft tissue texture was unacceptable because of insufficient keratinized tissue and vestibular depth. (b) Buccal donor site preparation. The donor site depicts a partial incision from tooth #3 to tooth #5. The graft was taken 2 mm away from the marginal gingiva of teeth #3, #4, and #5. (c) Buccal FGG fixation. The graft was placed on the recipient site near the mucogingival junction and secured with 6–0 nylon sutures by single interrupted sutures onto the FGG bed. (d) Two months follow‐up after the buccal FGG surgery.

After 2 months, both the vertical height and thickness of the mucosa surrounding the implant site exhibited an increase, resulting in a convex buccal contour. This enhancement in papillae growth facilitated the placement of an FP1 prosthesis. However, the previous flap advancement resulted in a lack of keratinized tissue and vestibular depth (Figure 3a). Therefore, we decided to perform FGG and harvest the graft from the buccal aspect of the premolar area to overcome the drawbacks of flap advancement and restore the color and texture of the area.

#### 2.1.2 Second Surgical Intervention (Buccal FGG)

The recipient bed was prepared with a partial thickness incision. The mobile mucosa was removed to reach the underlying dense connective tissue. The recipient bed was extended laterally from tooth #6 to tooth #8. The FGG was harvested from the buccal side of tooth #3 to tooth #5. A split‐thickness incision was performed from 2 mm apical to the gingival margin, including the keratinized attached gingiva and also 2–3 mm of alveolar mucosa. The graft was stabilized in the recipient site using a 6–0 nylon suture (single interrupted technique) (Figure 3b–d). The postoperative protocol included a nonsteroidal anti‐inflammatory drug (Ibuprofen 400 mg per pain) and rinsing with mouth rinse (0.12% chlorhexidine digluconate every 12 h for 14 days). After 2 weeks of uneventful healing, the sutures were removed.

#### 2.1.3 Third Surgical Intervention (Second Stage Implant Surgery)

**FIGURE 4:**
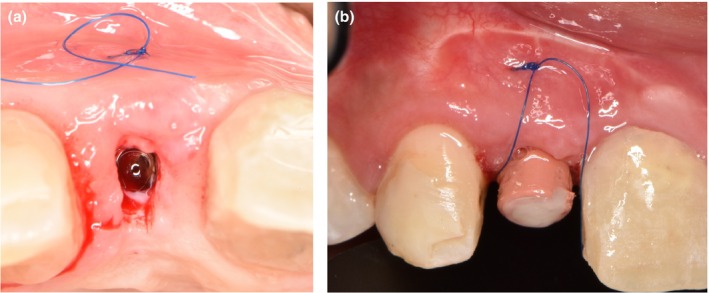
(a) Clinical view at 4 months; implant second stage surgery. A rolled flap from the palate was used to uncover the fixture and achieve buccal convexity. (b) Customized healing abutment. A customizable two‐piece NC healing abutment (Straumann) was fitted without pressing on papillae for optimal tissue growth.

**FIGURE 5:**
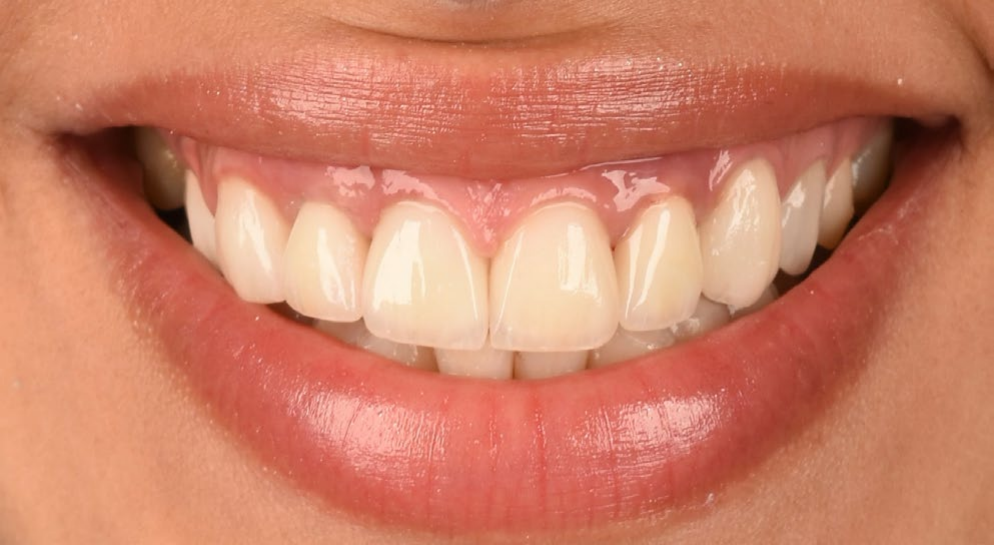
Seven months follow up; the increase of three millimeters in the height of keratinized tissue was observed. The final prosthetic crown was delivered and permanently cemented.

Two months later, the provisional restoration was removed and the implant was uncovered (Figure 4). The covering tissue was rolled under the tunneled buccal mucosa (Figure 4a). A customizable two‐piece NC healing abutment (Straumann) was installed without compressing the papillae tissue to allow maximum soft tissue augmentation without interference with the abutment (Figure 4b). During the following 3 months, the mesial and distal papillae were conditioned by adding composite material (Kerr) to the abutment to guide the papilla in the coronal direction. The addition of composite resin to the abutment was repeated until the excellent shape and growth of the interdental papillae were accomplished, and the papillae were filled and shaped in a sharp form. The final prosthetic phase was delivered when the desired papilla growth (3 mm) was observed (7 months after the implant surgery). After the final preparation of the gingiva using a round bur, the final restoration was cemented permanently (Figure 5).

## 3 Results and Conclusion

**FIGURE 6:**
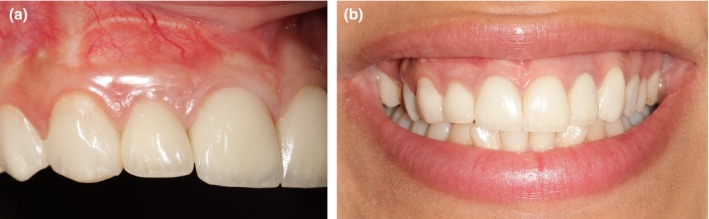
(a) Three years follow up; close up view. The pink esthetic score was measured at 14 as favorable. (b) Three years follow up; Frontal view. The mucosal margin was stable, also a favorable color and texture match was observed.

Seven months after the first surgery, an increase of 3 mm in the height of keratinized tissue was measured. In addition, the buccal contour and the papilla growth were acceptable (Figure 5). The prosthesis was delivered as FP‐1, making it hygienic and aesthetically appreciable. The surgical area had the same color and texture as the rest of the ridge, and the donor site healed without any issues. At 3 years follow‐up, the pink esthetic score [30] was measured at 14 (Figure 6a,b). The final clinical findings indicated the ultimate treatment success rate. Overall, the objective esthetic assessment was consistent with the patient's esthetic perception.

Given the limitations of this case report, it can be concluded that buccal FGG successfully achieved mucosal symmetry with the neighboring teeth regarding both color and texture while also reconstructing the mucogingival junction.

## 4 Discussion

Soft tissue augmentation utilizing connective tissue grafts in the esthetic zone can occasionally result in mucogingival defects. When the objective of soft tissue augmentation is solely to enhance the horizontal thickness or the buccal contour, employing tunneling techniques may reduce the necessity for significant flap advancement; consequently, minimizing scar formation [31, 32]. However, if soft tissue augmentation aims to enhance the papilla, significant flap advancement may be indicated. In this case report, following flap advancement intended to cover the connective tissue graft, a mucogingival defect was observed in terms of vestibular depth, as well as soft tissue color and texture. To address this issue, a buccal FGG was conducted. Several expert clinicians and researchers regarding soft tissue procedures have reported unfavorable outcomes following a classic FGG in the esthetic zone [22, 33]. A review article reported that FGG may lead to poor esthetic results in terms of color match and tissue texture, compared to the adjacent soft tissue [34].

To overcome the limitations of autogenous FGGs, xenogeneic/allogenic collagen matrices have been introduced as alternative materials [35]. McGuire et al. [36] reported that while sites grafted with FGG tended to retain the characteristics of the palatal tissue, sites that received living cellular constructs showed statistically significant superior esthetic results in terms of color match and texture.

In 2015, Urban et al. described two techniques [25, 27] to restore the severely distorted mucogingival complex in the esthetic zone. They used a xenogeneic collagen matrix [25] or a free connective tissue graft [27] in the coronal part and a strip of FGG in the apical extension of the recipient bed as a mechanical barrier for repositioning the mucogingival junction and deepening the vestibule. The results were aesthetically pleasing and also functional.

In 2020, they suggested using a strip of labial FGG, instead of a palatal one, to reduce the postoperative morbidity of the patients [26]. It was concluded that labial gingival graft with xenogeneic collagen matrix and connective tissue graft can be considered a viable approach to reconstruct the keratinized mucosa at implant sites with high patient satisfaction and esthetic outcome. A drawback of the combination of FGG and connective tissue graft is the increased patient morbidity and the need for two individual donor sites. Although the esthetic appearance is clinically better as compared to that of FGG [37], the histologic view showed the formation of scar tissue following the use of the collagen matrices [38, 39, 40]. Moreover, they may not be able to promote keratinized tissue neogenesis [39] and may be more prone to shrinkage, as compared to autogenous FGG [37, 41]. In the present case report, we utilized a non‐stripped buccal FGG and placed the graft in the marginal mucosa, compared to the apical aspect used in the strip technique introduced by Urban et al. [26]. Moreover, we included both the keratinized gingiva and the alveolar mucosa: 1‐to transfer the natural mucogingival junction to the recipient site for esthetic appearance and 2‐to widen the graft size to facilitate the suturing process.

Due to the limited donor site, the application of buccal FGG may not be feasible in cases with large areas of multiple implants. In addition, buccal FGG should be harvested carefully, especially in patients with a thin phenotype, to prevent the occurrence of fenestration or gingival recession in the donor site. Studies with large sample sizes are necessary to validate the results of this case report.

## Author Contributions

**Neda Moslemi:** conceptualization, methodology, project administration, supervision, writing – review and editing. **Mahdie Rahami:** investigation, writing – original draft, writing – review and editing. **Mohammad Shokri:** investigation, methodology, writing – review and editing. **Hossein Khoshkhou:** investigation, methodology, writing – review and editing. **Amir Raee:** conceptualization, methodology, writing – original draft, writing – review and editing.

## Ethics Statement

This case report was registered and verified by the ethical committee with the following registration number: IR.TUMS.DENTISTRY.REC.1403.047.

## Consent

Complete written informed consent was obtained from the patient for the publication of this study and the associated images.

## Conflicts of Interest

The authors declare no conflicts of interest.

## Data Availability

The data that support the findings of this study are available from the corresponding author upon reasonable request.
